# Oxidative Stress and Down Syndrome: A Route toward Alzheimer-Like Dementia

**DOI:** 10.1155/2012/724904

**Published:** 2011-11-29

**Authors:** Marzia Perluigi, D. Allan Butterfield

**Affiliations:** ^1^Department of Biochemical Sciences, Faculty of Pharmacy and Medicine, Sapienza University of Rome 00185 Rome, Italy; ^2^Department of Chemistry, University of Kentucky, Lexington, KY 40506, USA; ^3^Center of Membrane Sciences, University of Kentucky, Lexington, KY 40506, USA; ^4^Sanders-Brown Center on Aging, University of Kentucky, Lexington, KY 40506, USA

## Abstract

Down syndrome (DS) is one of the most frequent genetic abnormalities characterized by multiple pathological phenotypes. Indeed, currently life expectancy and quality of life for DS patients have improved, although with increasing age pathological dysfunctions are exacerbated and intellectual disability may lead to the development of Alzheimer's type dementia (AD). The neuropathology of DS is complex and includes the development of AD by middle age, altered free radical metabolism, and impaired mitochondrial function, both of which contribute to neuronal degeneration. Understanding the molecular basis that drives the development of AD is an intense field of research. Our laboratories are interested in understanding the role of oxidative stress as link between DS and AD. This review examines the current literature that showed oxidative damage in DS by identifying putative molecular pathways that play a central role in the neurodegenerative processes. In addition, considering the role of mitochondrial dysfunction in neurodegenerative phenomena, results demonstrating the involvement of impaired mitochondria in DS pathology could contribute a direct link between normal aging and development of AD-like dementia in DS patients.

## 1. Down Syndrome

Down syndrome (DS) is the most common genetic cause of mental retardation that arises from the triplication of the entire, or even part of chromosome 21 (trisomy21). Although the genetic alterations are responsible of the major clinical presentations of the disease such as craniofacial abnormalities, small brain size, accelerated aging, and cognitive defects, additional environmental factors seem to play an important role in determining the severity of multiple phenotypes. Genetic instability due to trisomy leads to the development of two types of phenotypes: (1) those present in every DS individual and (2) those that occur only in a subset of DS individuals. In addition, for any given phenotype there is considerable variability in expression that further results in a complex, “not-predictable” set of clinical signs [1]. For example, the extent of cognitive impairment in the DS population presents with a wide range of diversity. This wide variability may be explained, at least in part, by the “gene dosage hypothesis,” which states that some of the genes encoded on Chr21 are dosage sensitive—that is, three copies result in phenotypic effects—and contribute to the phenotypes of DS [2, 3]. This proposed scenario is further complicated by the fact that the abnormal expression of trisomic genes also affects disomic genes as well, which, in turn, are in part responsible of some clinical manifestations and ultimately results in as assembly of different DS phenotypes [4]. Thus, according to “the amplified developmental instability hypothesis”, the most important cause of the array of phenotypic features does not actually involve exclusively specific genes on Chr21 but rather elevated activity of sets of genes, regardless of their identity, which lead to a decrease in genetic stability or homeostasis [5] An interesting example of this effect is represented by the findings that newborns and children with DS are predisposed to a range of blood disorders, which include acute lymphoblastic leukaemia and acute megakaryocytic leukaemia (AMKL). In addition to trisomy 21, fetal haemopoietic progenitors acquire N-terminal truncating mutations in the key megakaryocyte-erythroid transcription factor GATA1 [6].

Improvements in quality of life of individuals with DS have resulted from improvements in medical care, identification and treatment of psychiatric disorders (such as depression, disruptive behavior disorders, and autism), and early educational interventions with support in typical educational settings [7, 8]. Also, largely owing to advances in medical care and attitude changes, the median age of death in this population has increased to 49 years, and the life expectancy of a 1-year-old person with Down syndrome is more than 60 years and is likely to improve [8].

Considering that DS patients presently have an improved life expectancy and quality of life, the comprehension of degenerative phenomena related to accelerated aging and neurodegeneration has received much attention from researchers. In fact, a link between the DS phenotype and an increased risk of the development of AD has now been firmly established [9]. The prevalence of dementia among DS patients is 8% in the age range 35–49, 55% in the age range 50–59, and 75% above the age of 60 years, but AD neuropathology is present in all of the cases by the age of 40 [10]. AD like dementia in DS population is characterized by the presence of senile plaques (SPs) and neurofibrillary tangles (NFTs) and by cholinergic and serotonergic reduction [11, 12]. However, although most DS patients have plaques early in life and even in the fetus, it is only very later on that they may develop AD. Thus, identification of common pathways together with specific differences of the neurodegenerative process occurring both in DS and AD currently represents an intense field of research. Among proposed hypothesis, oxidative stress is receiving much attention and may be considered a bridge between DS and AD.

## 2. Oxidative Stress (OS) in DS

**Figure 1:**
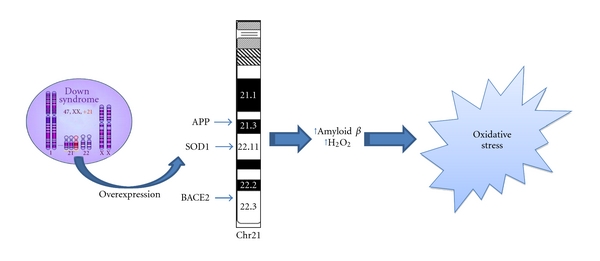
Oxidative stress and down syndrome. Increased conditions of oxidative stress are caused by the overexpression of some of the genes encoded by Chr21. Among these, amyloid precursor protein (APP), copper-zinc superoxide dismutase (SOD1), and beta secretase (BACE2) can directly or indirectly lead to OS.

Increasing number of studies have recently shown that OS occurs in DS pathogenesis and progression due to a deregulation of gene/protein expression associated with the trisomy characteristic of DS [13]. Increased production of ROS is also accompanied by mitochondrial dysfunction, which occurs in DS cells as early as from embryonic life [14]. Although oxidative stress implications in DS phenotype have been demonstrated [10, 11, 15–17], a direct cause-and-effect relationship between the accumulation of oxidatively mediated damage and clinical manifestation of DS is not yet strongly established. Growing evidence supports the occurrence of chronic oxidative injury in the brain that could imply a risk factor for subsequent neurodegeneration in aged DS patients [4, 18, 19]. Increased conditions of oxidative stress are caused by the overexpression of some of the genes encoded by Chr21 (Figure 1). Among these, one of the most relevant as a potential OS inducer is copper-zinc superoxide dismutase (SOD1). SOD1 is thought to have a major role in the first line of antioxidant defense by catalyzing the dismutation of O_2_^•−^ to molecular oxygen (O_2_) and H_2_O_2_, which can be converted by catalase (CAT) and by (selenium-containing) glutathione peroxidase (GPX) to water [20]. The triplication of Chr21, on which the SOD-1 gene is localized, leads to an imbalance in the ratio of SOD-1 to CAT and GPX, resulting in the accumulation of H_2_O_2_ [10]. Interestingly, all DS tissues, in addition to the brain, display an altered SOD-1/GPX activity ratio [21]. SOD-1 was found at levels approximately 50% higher than normal in a variety of DS cells and tissues, including erythrocytes, B and T lymphocytes, and fibroblasts. Indeed, the erythrocytes of DS children, adolescents, and adults exhibited systemic increases in SOD-1, SOD-1/GPX, or the SOD-1/(GPX + CAT) activity ratio. In addition, a decreased expression of peroxiredoxin 2 was detected in DS fetal brain which may contribute to enhanced susceptibility of DS neurons to free radical attack [22].

A crucial role of SOD-1 is further demonstrated by the study of Shin et al. which reported that transgenic mouse strains overexpressing wild-type human SOD1 (Tg-SOD1) showed to have mitochondrial swelling, vacuolization, or learning and memory deficits [23]. Mitochondrial ATP synthase alpha/beta chain and elongation factor Tu were aberrant in Tg-SOD1, while antioxidant proteins were found to be unchanged. Derangement of neuronal and mitochondrial proteins may indicate synaptosomal and neuronal loss in Tg-SOD1 hippocampus, already reported in morphological terms, and could help to understanding brain deficits in DS.

Consonant with the above-cited studies, Busciglio and Yankner [14] reported that neurons of DS patients exhibited a sharp increase in intracellular ROS which is also accompanied by elevated levels of lipid peroxidation. In addition, a proteomics study from Gulesserian et al. [24] showed that oxidative stress in fetal DS did not result from overexpression of SOD-1 protein but appeared to be the consequence of low levels of antioxidant enzymes involved in removal of hydrogen peroxide, such as glutathione transferases and thioredoxin peroxidases.

Interestingly, elevated levels of OS could also be caused by increased release of amyloid beta-peptide (A*β*). Many studies demonstrated that both A*β*(1-40/42) are able to induce OS [25–31]. Thus, the overexpression of the amyloid precursor protein (*APP*) gene, which is also encoded by Chr21, could explain in DS patients the overproduction of A*β* peptide, the major protein in SPs. Indeed, postmortem studies on DS brain evidenced accumulation of A*β*(1-42) peptide, a characteristic hallmark of AD pathology, which correlate with age [32]. Mehta et al. [33] found that A*β*(1-42) and A*β*(1-40) levels were higher in DS plasma than controls. The ratio of A*β*42/A*β*40 was lower in DS than in controls and a significant negative correlation between age and A*β*40 in DS and controls were observed, and between age and A*β*42 levels in DS but not in controls. Recently, a paper from the same group demonstrated that among adults with DS, decreasing levels of plasma A*β*42, a decline in the A*β*42/A*β*40 ratio, or increasing levels of A*β*40 may be sensitive indicators of conversion to AD, possibly reflecting compartmentalization of A*β* peptides in the brain [34]. However, recent studies from Anandatheerthavarada et al. [35] indicated that also full length APP itself may have deleterious effects, particularly targeting mitochondria. The authors proposed that under increased APP expression, a progressive accumulation of transmembrane-arrested APP caused perturbation of mitochondrial function, which in turn resulted in impairment of energy metabolism.

Moreover, mice overexpressing wild type human APP display cognitive defects and neuronal pathology similar to AD; these mice do not show significant A*β* deposition in the hippocampus [36]. In these mice, hAPP processing was basically nonamyloidogenic, while increased levels of phosphorylated tau in the hippocampus were observed. These findings support the notion that trisomy of APP may promote mitochondrial dysfunction in DS independent of aberrant A*β* deposition.

Related to APP metabolism, another gene encoded by Chr21 is the *β*-site APP-cleaving 2 enzyme (BACE2). BACE is homologous to BACE1, a *β*-secretase involved in the amyloidogenic pathway of APP proteolysis, and thus it has been hypothesized that the co-overexpression of both genes could contribute to Alzheimer's like neuropathology present in DS. However, co-overexpression of BACE2 and APP did not increase amyloid-*β* peptide concentration in brain of Tg mice. These results suggest that the in vivo effects of APP are not exacerbated by BACE2 co-overexpression but may have some protective effects in specific behavioral and cognitive domains in transgenic mice [37].

By mapping Chr21, another candidate gene that may be involved in OS is the enzyme carbonyl reductase (CBR). Carbonyls, which are cytotoxic metabolic intermediates, are detoxified by either oxidation catalyzed by aldehyde dehydrogenase (ALDH) or by reduction to their corresponding alcohols by carbonyl reductase (CBR) and/or alcohol dehydrogenase (ADH). Protein levels of both enzymes were found to be increased in several brain regions of both DS and AD patients because of enzyme induction by elevated carbonyls in DS and AD [38]. Further, carbonyl reductase is an oxidatively modified protein in brain of subjects arguably with the earliest form of AD, mild cognitive impairment [39].

There is evidence of a link between 1-carbon/transsulfuration (1C-TS) metabolism and DS. There are at least six genes encoding enzymes important for 1C-TS metabolism located on human Chr21, including the gene for cystathionine beta synthase (CBS) [40]. CBS catalyzes the condensation of serine and homocysteine to form cystathionine. It plays a critical role in linking the folate cycle and the methionine cycle and in regulating homocysteine levels [41]. In addition, CBS can convert cysteine to hydrogen sulfide, which researchers are beginning to recognize as an important neuromodulator in the brain [42]. There is evidence that CBS protein levels and enzyme activity are increased in persons with DS [43]. Elevated CBS activity can lower homocysteine levels, which in turn perturb the balance of 1C-TS metabolism and lead to elevated—perhaps toxic—levels of hydrogen sulfide. These metabolic alterations might play a role in the cognitive disability seen in DS [44, 45]. Accordingly, CBS is considered a risk factor for AD [46].

Another player in the complex C1-TS metabolism is also the trifunctional enzyme complex glycinamide ribonucleotide synthase-aminoimidazole ribonucleotide synthase-glycinamide formyl transferase (GARS-AIRS-GART), which catalyzes certain steps of *de novo* purine synthesis [47]. GART is aberrantly regulated and overexpressed in DS individuals and may be involved in the phenotype of DS [47]. Accumulation of uric acid, the end product of purine metabolism, is another feature of DS and there are some hypotheses about the pathogenetic mechanism leading to its increase [48]. Hyperuricemia has an interesting relationship with oxidative stress since it represents an important free radical scavenger and ROS themselves could influence its increase.

Chr21 also maps the gene for S100*β*, an astroglial-derived Ca^2+^-binding protein having neurotrophic role on neurons and glial cells. S100*β* is responsible to start up a gliotic reaction by the release of proinflammatory mediators, including nitric oxide and cytokines from microglia and astrocytes, which are, in turn, deleterious for neurons [49]. Interestingly, proinflammatory effect of S100*β* seems not to be restricted into the brain. Macrophages play a pivotal role in inflammatory diseases, occurring both in the brain and in the periphery. An aberrant S100*β* production has been observed in DS and AD [50]. It has been shown that S100*β* stimulates both NO production and iNOS protein transcription and expression in rat peritoneal macrophages [49].

Elevated OS has been demonstrated in peripheral and CNS specimens of DS patients and models thereof [14]. Increased levels of TBARS, total protein carbonyls, and advanced glycation endproducts (AGEs) in the cortex from DS fetal brain compared with controls were reported [51] and a marked accumulation of 8-hydroxy-2-deoxyguanosine (8OHdG), oxidized proteins and nitrotyrosine, in the cytoplasm of cerebral neurons in DS was found [52]. Elevated levels of isoprostane 8,12-*iso*-iPF2*α* (iPF2*α*), a specific marker of lipid peroxidation, have been measured in urine samples from adults with DS [25]. In addition, levels of AGEs, dityrosine, H_2_O_2_, and nitrite/nitrate were found to be significantly increased in urine samples of DS compared with age-matched controls [53]. These markers of oxidative damage were considered more consistent compared with 8-OHG, 15-F(2t)-IsoP, and TBARS which gave contrasting results. However, additional studies on large population are needed to confirm the reproducibility of these results.

The majority of OS data have been obtained by the analysis of animal models of the disease, including Ts65Dn mice and Ts1Cje mice. The Ts65Dn mouse carries a small chromosome derived primarily from mouse chromosome 16, causing dosage imbalance for approximately half of human chromosome 21 orthologs. These mice have cerebellar pathology with direct parallels to DS [54]. The Ts1Cje mouse, containing a translocated chromosome 16, is at dosage imbalance for 67% of the genes triplicated in Ts65Dn [55]. Ts1Cje mice do not express the SOD1 gene and show some DS-related abnormalities such as craniofacial alterations [56] and spatial learning deficits [57], but different from Ts65Dn mice.

Ishihara et al. [27] reported increased level of ROS and mitochondrial dysfunction in primary cultured astrocytes and neurons from Ts1Cje transgenic mice, confirming that the “gene-dosage” hypothesis is sufficient to explain, at least, the major part, of OS-induced intracellular damage observed in this animal model of DS. The authors also identified by redox proteomics approach the putative target proteins that were modified by lipid peroxidation-derived products [27]. ATP synthase mitochondrial F1 complex b subunit, *α*-enolase, and triosephosphate isomerase 1 were identified as proteins modified by 3-hydroperoxy-9Z,11E-octadecadienoic acid (13-HPODE). Neurofilament light polypeptide, internexin neuronal intermediate filament *α*, neuron specific enolase, peroxiredoxin 6, phosphoglycerate kinase 1, and triosephosphate isomerase were shown to be HNE-modified proteins. Thus, dysfunction of these proteins as a consequence of oxidative damage may affect ATP production, the neuronal cytoskeleton system, and antioxidant network function. Interestingly, previous redox proteomics studies from our laboratory previously found some of these proteins modified by hydroxynonenal, a reactive product of lipid peroxidation, in AD and MCI brain [39, 58, 59], suggesting that these brain proteins might contribute to cognitive dysfunction and neurodegenerative processes occurring in DS. These findings point out that DS and AD share common pathways of neurodegeneration that need to be further elucidated.

 In an effort to better understand the role of oxidative stress we have analyzed the amniotic fluid (AF) from women carrying DS pregnancy compared with that from women carrying healthy fetuses. While the majority of the studies have been performed on Down fetal brains or DS mouse model, few data are available on AF, which is a more reliable index of the physiological condition of the fetus. In analogy with CSF, which is considered “a window into the brain”, AF could be used for the identification of disease biomarkers to be coupled with current genetic screening. Thus, AF provides both physical and biochemical support for the developing fetus. Its composition is complex and includes fetal and maternal proteins, amino acids, carbohydrates, hormones, lipids, and electrolytes. Since AF is in direct contact with multiple organs of the fetus, AF contains high concentrations of proteins that are directly secreted from the fetus [60]. Not surprisingly, recent technological advances in proteomics have been actively utilized to investigate AF, in order to better understand its complex biological function and to discover disease-specific biomarkers for fetal aneuploidies and pregnancy-related complications. Once an abnormal proteomic profile is identified, it has to be compared with healthy closely matched controls, allowing for a disease-specific biomarker to be identified [61, 62].

Thus, we have evaluated a set of oxidative stress biomarkers in amniotic fluid from women carrying DS fetuses, and we found increased levels of oxidative stress, as indexed by increased protein oxidation, lipid peroxidation, reduction of GSH and Trx levels, and induction of the heat shock protein (HSP) response. By a redox proteomics approach, we have identified selective proteins that showed increased oxidation in DS AF compared with that from mothers carrying healthy fetuses. The identified proteins are involved in iron homeostasis (ceruloplasmin and transferin), lipid metabolism (Zinc-alpha2-glycoprotein, retinol binding protein 4 and Apolipoprotein A1), and inflammation (Complement C9, Alpha-1B-glycoprotein, Collagen alpha-1 V chain) with critical relevance in the clinical outcome of DS [63].

As previously mentioned, another important player in the oxidative stress hypothesis of neurodegeneration is A*β* peptide. Brain from DS subjects show consistent A*β* deposition and neurofibrillary tangle formation [64] that correlate with of age. Although plaque deposition is a very early event in DS patients, even in fetal development, it is only very later on that they may develop AD [65]. In fact, increased signs of dementia in DS after the age of 50 years appeared many years later the first signs of significant insoluble A*β* accumulation or plaque deposition and also after the first signs of neurofibrillary tangle pathology [66]. Thus, other factors, which may not directly link A*β* metabolism and tangles formation, have to be involved to cause consistent neuronal dysfunction and cognitive decline. Synaptic dysfunction may be a consequence of APP overexpression or increased A*β* [67]. Neuroinflammation [68], endosomal dysfunction [69], and oxidative damage [52] may play a crucial role in DS as well as in AD pathology [66].

Recent studies by our laboratory [17] were performed to establish an association between brain oxidative damage and A*β* neuropathology as a function of age in DS patients. Preliminary results showed that DS brains with neuropathological hallmarks of AD have more oxidative, but not nitrosative, stress than those with DS but without significant AD pathology, as compared with similarly aged-matched non-DS controls. Further studies are needed to better understand aging-related phenomena in DS, which from one side contribute to development of AD but also paradoxically result in AD neuropathology but without dementia.

The neuropathology of DS is complex and occurs with a wide variability. The characteristic hallmarks of neurodegenerative process are altered free radical metabolism and impaired mitochondrial function which both contribute to development of AD by middle age [70–72]. However, recent studies reported a quite surprising trend of oxidative stress damage in DS. While increased OS is detectable as early as during pregnancy [73] and increases over age in young DS, adults with DS do not show a significant increased oxidative damage to DNA [74]. These data could appear contradictory with other findings supporting the correlation of increased oxidative damage with increasing age. One of the reasons could be related to the samples analyzed, that is, peripheral lymphocytes, which indicate a cell-type with specific functionality and which could be able to activate compensatory mechanisms as the brain does not. In addition, different markers of oxidative stress do not always correlate with each other, because the ability of the cell to repair differently a “specific” damage in addition to different susceptibility of lipids, proteins, and nucleic acid to accumulates oxidative damage.

**Figure 2:**
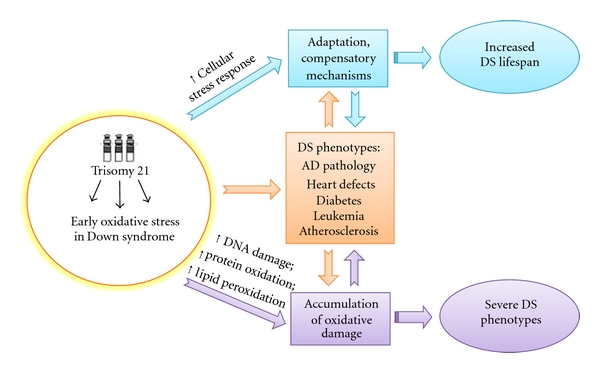
Putative adaptation to OS in down syndrome. OS occurs early in DS pathogenesis and progression. Accumulation of oxidative damage leads to severe phenotypes while the induction of compensatory mechanisms in response to chronic OS could result in “adaptation” and could contribute to improve the life span of DS subjects.

It seems likely that young DS experienced a sort of chronic oxidative stress and those “surviving” cells become more resistant by activating defense mechanisms that counteract increasing oxidative stress conditions over the lifespan [74]. This is reasonable by considering that newborn DS have to challenge with high levels of ROS that are responsible of the pathogenesis of many of the pathological manifestations. In contrast, this “experience” of OS promotes the survival of more resistant cellular phenotypes that show several dysfunctions (Figure 2). This hypothesis is further supported by studies from Head et al. [65], who showed by PET that compensatory increases in metabolic rate and activation of plasticity mechanisms in vulnerable brain regions in DS occurred prior to the development of dementia. The same genes, including APP, DYRK1A, SOD1, and RCAN1, which once overexpressed are responsible of impaired neuronal growth and synapse maintenance on the contrary may also promote the activation of compensatory mechanisms during aging [65].

For example, secreted forms of A*β*PP, in addition to be neurotoxic, can also function as neuroprotective factors [75] and possible cell adhesion molecules [76] and also play a role in cell signaling [77]. Interaction of A*β*PP with multiple protein networks might result in activation of complex compensatory responses. RCAN1 (regulator of calcineurin 1) has recently been shown to act synergistically with DYRK1A to impair the function of NFAT transcription factors which are involved in cell development. RCAN1 is highly expressed in neurons and overexpressed in DS brain [78]. Possible additional roles for RCAN1 include modulation of the chromosome 21 gene SOD1 [79] and playing a critical role in mitochondrial function [80]. It seems likely that some trisomic genes may interact with each other and are responsible of learning and memory deficit during development, but with increasing age their interaction may become beneficial and possibly protective [60]. The molecular mechanisms which drive dysfunction versus protection need to be extensively investigated. Based on these considerations, enhancing or supporting compensatory mechanisms in aging individuals with DS may be beneficial as suggested by intervention studies in animal models.

## 3. Mitochondrial Dysfunction in DS

Several reports have demonstrated that mitochondrial impairment plays a central role on neurodegeneration [16]. The first abnormalities of mitochondrial function (abnormal shape, reduced levels of microtubules, etc.) was observed in cultured cerebellar neurons from trisomy 16 (Ts16) mice [81]. Previous findings demonstrated deficient functionality of mitochondrial enzymes, including monoamine oxidase, cytochrome oxidase, and isocitrate dehydrogenase [82].

Numerous studies have demonstrated that the accumulation of mitochondrial DNA (mtDNA) mutations is a major contributor to degenerative diseases and human ageing [83]. Studies from several groups suggested that mtDNA mutations have a role in the pathogenesis of DS [71, 84, 85]. Apart from helping to explain free radical damage and development of AD, the presence of mtDNA mutations could explain the association of DS with premature ageing and diabetes [86]. Mutations in mtDNA may bring about an increase in the generation of free radicals and reduce ATP levels. This, in turn, could affect the synaptonemal complex and chromosome segregation, also alter recombination and so lead to aneuploidy.

Druzhyna et al. [87] demonstrated not only an increase of mtDNA oxidative damage but also a reduced functionality of specific repair systems in fibroblasts from DS patients. Increased oxidative damage was a consequence of increased superoxide formation that was demonstrated in Ts16 neurons compared to control neurons. This condition persisted also in the presence of rotenone, a mitochondrial respiratory chain complex I inhibitor, which was able to block O_2_^•−^ production in diploid neurons, but not in Ts16 neurons. This different behavior between Ts16 neurons and diploid neurons also was evident when cells were treated with carbonyl cyanide p-trifluoromethoxyphenylhydrazone, used to uncouple mitochondrial oxidative phosphorylation, which caused irreversible deficiency in the energy metabolism in Ts16 neurons, but not in diploid control neurons. Thus, an increased O_2_^•−^ basal generation in Ts16 neurons results from deficient complex I coupled with an impaired mitochondrial energy metabolism that ultimately leads to neuronal cell death [88]. A selective impairment of complex I activity was demonstrated in isolated cortex mitochondria from Ts16 mice by administration of its normal substrates, malate, and glutamate, but not with the Complex II substrate succinate [89]. Accordingly, a very recent paper by Valenti et al. [90] reported a selective deficit in the catalytic efficiency of Complex I in DS-HSFs (Down's syndrome human fetal skin fibroblasts). The Complex I deficit was associated with a decrease in cAMP-dependent phosphorylation of the 18 kDa subunit of the complex, due to a decrease in PKA (protein kinase A) activity related to reduced basal levels of cAMP. Furthermore, the authors measured a 3-fold increase in cellular levels of ROS, in particular O_2_^•−^, mainly produced by DS-HSF mitochondria. This effect was prevented by dibutyryl-cAMP, a membrane-permeable cAMP analogue, suggesting its involvement in ROS production.

H_2_O_2_ production and calcium uptake did not differ significantly in the Ts16 mitochondria, while a decrease in pyruvate dehydrogenase levels was detected, similar to the pattern found in Parkinson's disease [91].

Further details on mitochondrial functionality were underscored by a study by Conti et al. [13]. The authors analyzed the expression profile of several genes located on Chr21 using oligonucleotide microarrays in hearts of human DS fetuses compared with normal fetuses. The authors concluded that dosage-dependent upregulation of Chr21 genes causes dysregulation of the genes responsible for mitochondrial function and for the extracellular matrix organization in the fetal heart of trisomic subjects [13].

Direct evidence for an in vivo alteration of mitochondrial function in blood cells from DS patients was reported by Roat et al. [92], who found an increased loss of ΔΨ(m), underlying the presence of an increasing susceptibility of these organelles to damaging agents. As noted above, mitochondrial function is also regulated by the methyl status due to the presence on Chr21 of the gens for specific CBS, which participates in recycling of methionine/homocysteine in the methyl cycle sequence of reactions. In fact, methylation is a necessary event in mitochondria and relies on the availability and uptake of the methyl donor S-adenosylmethionine. Indeed mitochondrial dysfunctions have been widely described in DS, but they have never been correlated to a possible mitochondrial methyl unbalance. Infantino et al. [93] recently showed that the mitochondrial levels of S-adenosylmethionine were reduced in DS compared to control cells consistent with a methyl imbalance on mitochondria functionality.

## 4. Concluding Remarks

Within the context of the reported findings discussed above, we hypothesize that trisomy affects gene/protein expression that results in increased OS conditions and impaired mitochondrial function. These alterations occur early in DS as demonstrated by studies performed on fetal brain and amniotic fluid from DS pregnancy and play an important role in neurodegeneration.

Several studies suggested different mechanistic causes for the changes in redox state in contributing to early neural pathological changes in DS brain. OS conditions arise not only from overexpression of SOD1 but also as a consequence of low levels of reducing agents and antioxidant enzymes. Redox imbalance is further affected by overproduction of A*β*, which accumulates into plaques across the lifespan in DS as well as in AD. A*β* toxicity has been shown to be a major effector of neuronal loss and cognitive dysfunctions observed both in DS and in AD and contributes to exacerbate oxidative damage into the brain. In fact, OS is a crucial factor because it affects multiple pathways related to cell growth/death, gene expression, and protein function, among many others. It is now well accepted that OS contribute to neurodegeneration, but in the case of DS and AD, genetic similarities, due to the fact that some of the genes responsible for familial form of AD are encoded by Chr21, provide an interesting field of research for the comprehension of many yet unsolved issues.

Based on this notion, it is possible that using antioxidant nutrients to scavenge oxygen-derived free radicals may modulate some of the complications of DS. A very recent paper by Lott et al. [94] demonstrated that a 2-year randomized, double-blind, placebo-controlled trial with daily oral antioxidant supplementation (900 IU of alpha-tocopherol, 200 mg of ascorbic acid, and 600 mg of alpha-lipoic acid) was effective, safe, and tolerable for individuals with DS and dementia. However, individuals receiving the antioxidant supplement showed neither an improvement in cognitive functioning nor a stabilization of cognitive decline compared with control group.

These data are in contrast with those obtained by Lockrow et al. [95], who treated Ts65Dn mice with a long-term antioxidant supplementation. Supplementation with vitamin E effectively reduced the levels of ROS in the adult Ts65Dn brain. Also, Ts65Dn mice receiving vitamin E exhibited improved performance on a spatial working memory task and showed an attenuation of cholinergic neuron pathology in the basal forebrain.

This discrepancy likely results from the “biological gap” between human and animal studies. Though transgenic mice are a useful model to study the molecular basis of a disease and test the efficacy of drug treatment, they do not show all the features of human disease. In particular, when testing the protective effects of antioxidants, supplementation has to be initiated before persistent oxidative damage occurs. For example, many individuals with AD most likely have significant AD pathology by the time of diagnosis. This phenomenon, reasonably, is one of the major limits of clinical trials that should be taken into account, such that antioxidants should be administered as putative modulators of disease at the very early stage of the disease.

Although limits of antioxidant therapies exist, an intriguing prospective could be offered by the comprehension of putative compensatory mechanisms which are activated even in the presence of genetic instability in the DS population that could play a role in explaining the wide variability of phenotypes. In fact, although overexpression of several genes on Chr21, including APP, DYRK1A, SOD1, and RCAN1, lead to impaired neuronal growth and synapse maintenance, at the same time the same genes may also induce an adaption through the action of compensatory pathways during aging. DS may represent an informative model of prodromal AD; thus, promising results may be available by the analysis of DS brain or brain from DS-relevant transgenic mice. Such studies are ongoing in our laboratories.

## Abbreviations

DS: Down syndrome
AD: Alzheimer disease
MCI: Mild cognitive impairment
SP: Senile plauques
NFT: Neurofibrillary tangles
A*β*: Amyloid beta peptide
SOD1: Cu-Zn superoxide dismutase
CAT: Catalase
GPX: Glutathione peroxidase
CBS: Cystathionine beta synthase
APP: Amyloid precursor protein
OS: Oxidative stress
Chr21: Chromosome 21
BACE: *β*-site APP-cleaving enzyme
GATA1: Megakaryocyte-erythroid transcription factor
RCAN1: Regulator of calcineurin1
DYRK1A: Dual-specificity tyrosine(Y)-phosphorylation-regulated kinase 1A.
